# Microbes, metabolites and (synaptic) malleability, oh my! The effect of the microbiome on synaptic plasticity

**DOI:** 10.1111/brv.12812

**Published:** 2021-11-03

**Authors:** Ayala Glinert, Sondra Turjeman, Evan Elliott, Omry Koren

**Affiliations:** ^1^ Azrieli Faculty of Medicine Bar Ilan University 8 Henrietta Szold Safed 1311502 Israel

**Keywords:** microbiome, central nervous system (CNS), enteric nervous system (ENS), neurons, synaptic plasticity, long‐term potentiation (LTP), neurotransmitters, immune system

## Abstract

The microbiome influences the emotional and cognitive phenotype of its host, as well as the neurodevelopment and pathophysiology of various brain processes and disorders, *via* the well‐established microbiome–gut–brain axis. Rapidly accumulating data link the microbiome to severe neuropsychiatric disorders in humans, including schizophrenia, Alzheimer's and Parkinson's. Moreover, preclinical work has shown that perturbation of the microbiome is closely associated with social, cognitive and behavioural deficits. The potential of the microbiome as a diagnostic and therapeutic tool is currently undercut by a lack of clear mechanistic understanding of the microbiome–gut–brain axis. This review establishes the hypothesis that the mechanism by which this influence is carried out is synaptic plasticity – long‐term changes to the physical and functional neuronal structures that enable the brain to undertake learning, memory formation, emotional regulation and more. By examining the different constituents of the microbiome–gut–brain axis through the lens of synaptic plasticity, this review explores the diverse aspects by which the microbiome shapes the behaviour and mental wellbeing of the host. Key elements of this complex bi‐directional relationship include neurotransmitters, neuronal electrophysiology, immune mediators that engage with both the central and enteric nervous systems and signalling cascades that trigger long‐term potentiation of synapses. The importance of establishing mechanistic correlations along the microbiome–gut–brain axis cannot be overstated as they hold the potential for furthering current understanding regarding the vast fields of neuroscience and neuropsychiatry. This review strives to elucidate the promising theory of microbiome‐driven synaptic plasticity in the hope of enlightening current researchers and inspiring future ones.

## I. INTRODUCTION

The microbiome–gut–brain axis refers to the way the resident gut microbiome communicates with and impacts the central nervous system. It has been firmly established (Ma, Forney & Ravel, 2012; Zarco, Vess & Ginsburg, 2012; Shreiner, Kao & Young, 2015; Tognini, 2017; Stiemsma & Michels, 2018; Altmäe, Franasiak & Mändar, 2019; Binyamin *et al*., 2020) that the microbiome is critical for normal physiological development and maintenance of homeostasis in all major multi‐organ systems of the body, and any significant perturbations to the microbiome may lead to a host of severe disorders (Shamriz *et al*., 2016; Seo, Boros & Holtzman, 2019; Goldberg *et al*., 2020; Shouval *et al*., 2020; Xavier *et al*., 2020; Zhang *et al*., 2020). Although the terms ‘microbiota’ and ‘microbiome’ are often used interchangeably, there is a small but important distinction. ‘Microbiota’ refers to the ecosystem of microorganisms in a given physiological environment, and includes colonizing entities such as bacteria, archaea, viruses, and eukaryotes, whereas ‘microbiome’ refers to the entirety of the microorganisms themselves along with their genetic material. This review focuses on the bacterial constituents of the gut microbiome (other constituents of the gut microbiome are not within the scope of this review).

Evidence links the microbiome to numerous phenomena (Leung & Thuret, 2015; Tognini, 2017; Strandwitz, 2018; Cryan *et al*., 2019) in the central nervous system (CNS), such as learning and memory, mental and emotional wellbeing and neuropsychiatric disorders, but the exact means by which these phenomena are shaped remains unclear. We propose that the microbiome affects the CNS *via* processes that contribute to synaptic plasticity in the CNS as well as the enteric nervous system (ENS), two systems that are engaged in constant bidirectional communication.

The ENS (Sasselli, Pachnis & Burns, 2012; Hyland & Cryan, 2016) is composed of two ganglionated plexi which facilitate the autonomic functionality of the gut. They do so independently of extrinsic sympathetic or parasympathetic inputs, earning the nickname ‘the second brain’. Although the ENS is a subsection of the peripheral nervous system, it is structurally similar to the CNS. Its various neuronal cell types are organized in a complex circuitry which orchestrates the integration of stimuli and induction of reflexes, enabling proper gut function. All of the major neurotransmitters that regulate neuronal processes in the brain also have vital enteric roles and are abundantly present in the gut (Mittal *et al*., 2017). Recent work has linked ENS dysfunction to the pathophysiology of diseases such as Parkinson's (Parashar & Udayabanu, 2017), Alzheimer's (Seo *et al*., 2019) and autism spectrum disorder (ASD) (Mulle, Sharp & Cubells, 2013), hitherto exclusively related to the CNS. The close association between the microbiome (Sasselli *et al*., 2012; Rao & Gershon, 2016) and the ENS suggests that the former plays a role in these devastating disorders.

Aside from regulating and maintaining the complex functionality of the gut, the enteric nervous system sends constant homeostatic feedback to the brain *via* the vagus nerve (Sasselli *et al*., 2012; Mittal *et al*., 2017). It is the longest parasympathetic nerve in the body. Not only does it enervate many organ systems, but it is also comprised mostly of afferent fibres carrying sensory information from the gut back to the brain. Vagal signalling, along with the hypothalamic–pituitary–adrenal axis (HPA) (Herman, 2018) and the immune system, constitute the bidirectional gut–brain axis. Since the microbiome has been shown to interact with and affect all three of these pathways (vagal signalling, HPA axis, immune system), it is well established that they are all crucial to the maintenance of the microbiome–gut–brain axis (Foster & McVey Neufeld, 2013; Rea, Dinan & Cryan, 2016; Strandwitz, 2018; Cryan *et al*., 2019; Frankiensztajn, Elliott & Koren, 2020).

Moreover, these three systems are tightly interconnected by several regulatory feedback mechanisms (Kabouridis & Pachnis, 2015; Obata & Pachnis, 2016; Fung, Olson & Hsiao, 2017; Yoo & Mazmanian, 2017; Kim, 2018). Vagal nerve endings present in the gut detect immunological mediators triggered by the microbiome and are then able to modulate the release of pro‐inflammatory cytokines by stimulating the release of acetylcholine (Ach). Additionally, vagal afferents are the conduit by which various microbial metabolites reach the brainstem solitary tract nucleus, which in turn projects to the hypothalamus and influences the HPA axis. The HPA axis is regarded as the body's primary stress‐response mechanism, whereby a cascade of signals between the hypothalamus, pituitary gland and adrenal cortex mediates glucocorticoid release, which initiates a metabolic and neuromodulatory stress response in the brain.

The mechanistic interface (Geuking *et al*., 2014; Kim, 2018; Zheng, Liwinski & Elinav, 2020) with the microbiome is distributed among the immune and enteric nervous systems and the HPA axis. Microbial metabolites engage the mucosal immune system and enteric nerve endings, both of which interact with one another, as well as the HPA axis. All of these, in turn, communicate information back to the CNS, which integrates the various stimuli and adapts accordingly. Moreover, there is growing evidence suggesting that the key to microbial involvement in host homeostasis lies within the microbial metabolites secreted into the gut and their interactions with various host pathways, such as those mentioned above.

A key aspect of neuronal tissue is its ability to adapt, physically as well as functionally, to a highly dynamic influx of stimuli and incorporate these adaptations into long‐lasting alterations in signal transduction and information retention. This is achieved by synaptic plasticity (Kotaleski & Blackwell, 2010; Bai & Suzuki, 2020), one of the most fundamental aspects of the nervous system, and seen in species ranging from flies to humans. It is worth mentioning that long‐term potentiation (LTP) and long‐term depression (LTD) are believed to only be cellular correlates of the natural processes we refer to as learning and memory and that the inductive properties are experimentally inferred. We believe that plasticity is an essential component of the microbiome–gut–brain axis because of its central role in modifying the CNS, ENS and HPA axis, as well as the immune system (Levy & Tasker, 2012; Cortés‐Mendoza *et al*., 2013; Maguire, 2014; Kabouridis & Pachnis, 2015).

Synaptic plasticity is the ability of neuronal tissue to undergo physical and functional changes at the level of individual connections between neurons (Citri & Malenka, 2008). Hebb was the pioneer behind the idea that resonating neuronal transmission among a transitory cellular repertoire lays the groundwork for memory formation. This theory assumes a fundamental adjustment in the firing rates of the participating synapses which takes place in direct correlation with the activity level in a given synapse (Hebb, 2002).

These changes, which are typically long‐lasting, occur in response to fluctuations in stimuli, allowing the host the ability to adapt to and thrive in dynamic environments. Quite simply, synaptic plasticity is cardinal to appropriate neuronal functionality. It is the cornerstone of learning, memory consolidation, emotional regulation, stimulus integration into behavioural adaptations and homeostatic maintenance of the brain and ENS. The various processes underlying these changes to synaptic architecture and transmission are induced primarily by LTP or LTD. Colloquially explained as ‘neurons that fire together wire together’, based on the ground‐breaking work of Hebb (2002), LTP refers to the strengthening of synapses, both physically and in terms of signal propagation capacity, as a result of high‐frequency stimuli. LTD (Collingridge *et al*., 2010) refers to a weakening of synaptic connectivity as a result of low‐frequency stimuli. LTP was initially observed in the hippocampus (Kumar, 2011), but observations have since been recorded in many other major areas of the brain. In studying synaptic plasticity, emphasis is given to areas of the brain that are known hubs of activity with projections to many other functional regions, especially structures that are implicated in learning and emotional regulation.

Synaptic plasticity (Glanzman, 2010; Kotaleski & Blackwell, 2010; Chater & Goda, 2014) is controlled by a combination of genes expressed *via* various signalling pathways, post‐translational modification of proteins, trafficking of receptors, and alterations to ion channel permeability. The revolutionary work of Kandel & Schwartz (1982) on the molecular mechanisms of synaptic plasticity corroborated Hebb's hypothesis and laid the groundwork for contemporary research.

Whilst the majority of work on the significance of synaptic plasticity has been conducted in the CNS, emerging evidence shows the importance of ENS plasticity. ENS‐mediated functions of the gut, including motility, hormone secretion and chemo‐sensitivity, have been shown to be altered following exposure to different nutrients and hormones, prolonged stress or inflammation. Although the exact mechanisms by which enteric neural tissue changes over the course of a lifetime remain to be elucidated, modulations in the ENS were shown to occur in response to specific nutrients, as well as peptide hormones secreted in the stomach and gut and sex hormones. Examples of these modulations include the hyper‐excitability of enteric neurons following an inflammatory response, altered action potential frequency across populations of neurons and lowered basal membrane potential (Mawe, Strong & Sharkey, 2009).

Given the increased importance attributed to ENS plasticity (Mawe *et al*., 2009; Schaefer, Van Ginneken & Copray, 2009), and in light of its implication in neurological disorders and the constant bidirectional communication with the CNS, we believe that the microbiome affects behaviour, cognition and emotional regulation by influencing plasticity in both the ENS and the CNS.

The aim of this review is to examine the microbiome–gut–brain axis in the context of synaptic plasticity, focusing on how the microbiome affects various plasticity‐inducing factors, including neurotransmitters, immune mediators and more.

The myriad ways in which the microbiome influences the ENS and CNS can be linked, to a great extent, to synaptic plasticity, both directly and indirectly (*via* mechanisms that contribute to synaptic plasticity). Therefore, we hypothesize that the most significant mechanistic relationship between the microbiome and the emotional, behavioural and cognitive phenotypes of the host is based on the microbiome's contribution to induction and maintenance of synaptic plasticity.

Our review provides initial support for this hypothesis and identifies mechanisms for further research through a discussion of potential research directions necessary to elucidate the connection between the microbiome and synaptic plasticity in the ENS and CNS. Understanding the mechanistic interaction between the microbiome and plasticity is of prime importance because of synaptic plasticity's involvement in severe neuropsychiatric disorders such as schizophrenia, ASD, bipolar disorder and depression. Relevant literature was sourced using the following search pattern: after searching for “synaptic plasticity” and becoming acquainted with the relevant terminology, each focal term was queried together with "microbiome", e.g “microbiome + synaptic plasticity”, “microbiome + LTP”, “microbiome + calcium signalling”, etc.

## II. MECHANISMS OF SYNAPTIC PLASTICITY

An in‐depth review of the many aspects of synaptic plasticity is beyond the scope of this paper. Briefly, there are several types of plasticity associated with the frequency of stimuli received at the neuron, the brain region and the type of cells involved. Here we focus on plasticity‐associated mechanisms and signalling pathways that have been shown to be modulated by the microbiome, including neurotransmitters (De Vadder *et al*., 2018; Jadhav *et al*., 2018), calcium signalling (Engevik *et al*., 2019; Schalla & Stengel, 2020) and immune mediators (Schirmer *et al*., 2016; Fung *et al*., 2017; Kim, 2018; Xue *et al*., 2018; Cerovic, Forloni & Balducci, 2019). Additionally, we focus mainly on LTP because it is studied more robustly and is easily observed phenotypically, especially in terms of microbial involvement in its induction.

Plasticity is induced through neurotransmitters that are released from the presynaptic neuron and the postsynaptic cell (Goto, Yang & Otani, 2010; Korb & Finkbeiner, 2011; Lesch & Waider, 2012; Leal, Comprido & Duarte, 2014; Lu, Nagappan & Lu, 2014; Maguire, 2014; Pribiag & Stellwagen, 2014; Woolfrey & Dell'Acqua, 2015; Nanou & Catterall, 2018; Limanaqi *et al*., 2019). The terms ‘excitatory’ and ‘inhibitory’, as they pertain to neurotransmitters and synapses, refer to the alteration in the resting membrane potential of the postsynaptic cell and the subsequent probability of firing an action potential. Glutamate is the main excitatory neurotransmitter in the CNS, and its main target ionotropic receptors are the N‐methyl‐D‐aspartate receptor (NMDAR), α‐amino‐3‐hydroxy‐5‐methyl‐4‐isoxazolepropionic acid (AMPAR) and kainate. These receptors are crucial to plasticity processes, and neuropsychiatric disorders emerge in their absence (Rao & Finkbeiner, 2007; Zarate & Manji, 2008). Calcium, which enters the cell after activation of NMDARs, is fairly unique in that it triggers an elaborate signalling cascade that results in phosphorylation (and consequent increased expression) of AMPARs by Ca^2+^‐calmodulin‐dependent protein kinase 2 (CAMKII). Gamma‐aminobutyric acid (GABA) is the most abundant inhibitory neurotransmitter and has been shown to induce either LTP or LTD, according to the varying postsynaptic signalling cascades recruited by the different GABAergic receptor subunits (Jappy *et al*., 2016). Other neurotransmitters, such as serotonin (5‐HT) and dopamine, are also involved in plasticity‐related mechanisms, due to their wide distribution across the brain and their implication in a wide array of complex cognitive and emotional processes.

Contrary to the decades‐long belief that immune cells are only present in the brain during severe pathological situations (D'Agostino *et al*., 2012), it is now known that several immune mediators, under tightly controlled regulation, are necessary for homeostatic brain processes, including plasticity (Levin & Godukhin, 2017). The immune system is also functionally prominent in the ENS, where, in conjunction with all the neurotransmitters known to operate in the brain, it mediates many aspects of ENS functionality (Sasselli *et al*., 2012; Yoo & Mazmanian, 2017). The main branches of the gut–brain axis are comprised by numerous molecular and genetic pathways that are known to contribute to the induction and maintenance of synaptic plasticity.

## III. EVIDENCE OF MICROBIAL EFFECTS ON PLASTICITY IN THE ENS AND CNS

Germ‐free (GF) animals are raised in a completely sterile environment, allowing them to serve as a platform for microbial transplants towards understanding causative effects of microbiome and host state on one another. While GF mice offer a blank slate for such studies, there is accumulating evidence that the GF condition is implicated in a range of health complications across the lifespan, which could make interpretation of findings more challenging (Dominguez‐Bello *et al*., 2019).

There is substantial evidence of altered behaviour, cognition and mood, in GF mice (Bercik *et al*., 2011; McVey Neufeld *et al*., 2015; Sarkar *et al*., 2020; Manca *et al*., 2020), indicating a microbial contribution to synaptic plasticity, as these phenotypic phenomena are rooted mechanistically in synaptic plasticity. GF mice also exhibit an altered neurophysiological transcription profile (Hölzel *et al*., 2010; Gareau *et al*., 2011; Heijtz *et al*., 2011; Ho, Lee & Martin, 2011; Lu *et al*., 2018), particularly in areas known to be highly involved in learning processes, such as the hippocampus and amygdala. Specifically, expression of immediate early genes (IEGs), which are directly involved in induction of plasticity, is markedly different in GF mice (Hoban *et al*., 2018). Evidence suggests that hippocampal plasticity in GF mice is regulated in a sex‐dependent manner (Darch *et al*., 2021). Due to the practical difficulty of conducting electrophysiological studies on GF mice, behavioural testing is often used as a proxy for altered plasticity (Medendorp *et al*., 2018). Young GF mice transplanted with the microbiome of aged mice exhibited a decrease in plasticity and in expression of neurotransmission‐related genes in the hippocampus, alongside marked cognitive deficits (D'Amato *et al*., 2020). The endocannabinoidome is a complex signalling pathway which has been linked to synaptic plasticity (Xu & Chen, 2015), learning and memory, behavioural adaptations and neurogenesis (Prenderville, Kelly & Downer, 2015). Manca *et al*. (2020) found the endocannabinoidome pathway to be impaired in GF mice, offering support to the hypothesis that it underlies some of the cognitive and behavioural differences in GF mice compared to specific‐pathogen‐free (SPF) mice. GF mice exhibit markedly altered behavioural and cognitive phenotypes, in part due to altered expression of brain‐derived neurotrophic factor (BDNF) and NMDARs, implicated in depression (BDNF) and neurogenesis and synaptic plasticity (NMDARs) (Neufeld *et al*., 2011). GF mice also have different levels of neurotransmitter secretion (Strandwitz, 2018), such as 5‐HT, dopamine and Ach, which were shown to influence plasticity in the brain. Several research groups have demonstrated the effects of antibiotics on behavioural phenotypes and expression profiles of key plasticity‐related compounds in the brain. Plasticity in the ENS is exemplified by increased levels of sensory sensitivity in the gut (Schaefer *et al*., 2009), induced by the microbiome (van Thiel *et al*., 2020). GF animals and those given antibiotics have altered gut neuronal transmission, coupled with a difference in membrane potential, either basally or following the firing of a neuronal signal (Carabotti *et al*., 2015). Calcium signalling is crucial in neuronal transmission and the induction of plasticity, and the microbiome has been shown to moderate the expression of calbindin, a calcium‐binding protein (McVey Neufeld *et al*., 2015).

*Lactobacillus reuteri* (Kunze *et al*., 2009) has been shown to alter the activity of calcium‐dependent channels in a subpopulation of enteric sensory neurons which are implicated in intestinal‐disorder‐derived pain. Rats fed an *L. reuteri* probiotic exhibited reduced levels of calcium‐dependent potassium channels, which lead to a lowered threshold for action‐potential firing, and consequently increased neuronal excitability. Another study (Perez‐Burgos *et al*., 2015) into the possible role of *L. reuteri* in amelioration of enteric pain sensations examined the effects of probiotic supplementation on capsaicin‐induced activation of transient receptor potential vanilloid 1 (TRPV1) channels. These channels are thought to mediate the transmission of nociceptive signals, and capsaicin is a known irritant which activates these channels in a manner leading to increased intracellular calcium currents. Mice treated with *L. reuteri* exhibited a reduced capsaicin‐induced excitatory response, accompanied by decreased TRPV1 expression.

There is thus a growing body of evidence that links the microbiome to the induction and maintenance of synaptic plasticity in both the ENS and CNS, suggesting that this crosstalk may be a key component of the microbiome–gut–brain axis.

## IV. MICROBIOME–GUT–BRAIN AXIS – FROM THE GUT EPITHELIUM TO THE BRAIN AND BACK

**Fig. 1:**
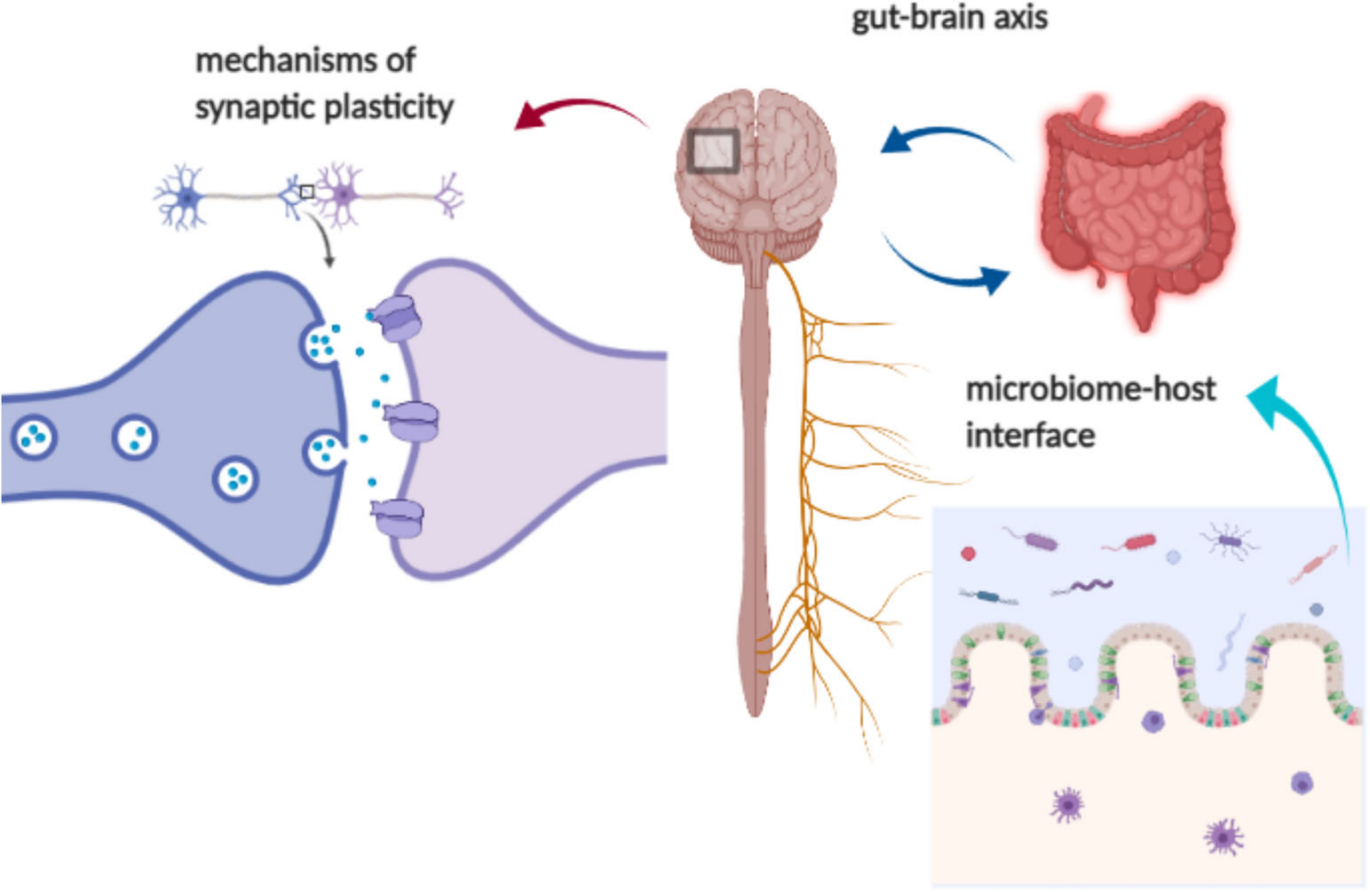
A general overview of the microbiome–gut–brain axis. The proposed mechanism by which the microbiome exerts its influence on the central nervous system is comprised of several elements. The first is interaction with the microbiome–host interface, the gut epithelium and enteric nervous and immune systems, which then synapse with the central nervous system *via* the vagus nerve and systemic immune interactions. The vagus is hypothesized to be a conduit for microbial metabolites directly to the brain, where, on a synaptic level, they influence the various mechanisms of synaptic plasticity.

The gut epithelium is the site of initial interaction between the resident microbiome and the host (Kabouridis *et al*., 2015; Okumura & Takeda, 2017; Solis *et al*., 2020). The physiological environment of the gut encompasses well‐developed neurological, immune and endocrine systems. The ENS is an autonomic neuronal network which communicates bi‐directionally with the CNS and autonomic nervous system (ANS) by way of both sympathetic and parasympathetic fibres synapsing in the gut. Enteric neurons express mechanoreceptors, chemo‐sensors and other signals which transmit a wide array of sensory, motor, molecular and vasodilator information back to the brain *via* the vagus nerve. The microbiome is thought to influence the neuronal activity of the ENS and CNS by secretion of neuromodulatory molecules (Fig. 1; Cryan & O'Mahony, 2011; Tognini, 2017; Strandwitz, 2018). Dopamine, noradrenaline (NA), 5‐HT, GABA and Ach are known to be produced by a wide array of bacteria (Strandwitz, 2018). Additionally, the gut microbiome was shown to play a significant role in the metabolism of tryptophan (Gao *et al*., 2020), which is the precursor of 5‐HT synthesis by the host. This is important because the majority of the body's 5‐HT is synthesised and utilized in the gut, with evidence of a constant feedback loop with 5‐HT synthesis in the brain.

Besides being the largest sensory organ, the gut is also the largest immune organ in the body, with the densest population of immune cells and molecular mediators (Yoo & Mazmanian, 2017). The mucosal immune system is highly specialized in its recognition of and response to pathogenic antigens (Carabotti *et al*., 2015; Rao & Gershon, 2016; Yoo & Mazmanian, 2017). Early‐life microbial colonization is thought to mediate maturation of the immune system, as shown in GF mice. Examples of microbiome–immune system interactions include microbial regulation of immune cell migration, differentiation and function of innate immunity constituents, induction of inflammation mediators (Geuking *et al*., 2014) and cytokine secretion modulation (Schirmer *et al*., 2016) by microbial short‐chain fatty acid production (Patel *et al*., 2012). Microbial perturbations are also linked to severe immunological disorders, including autoimmune diseases (Zhang *et al*., 2020).

The mucosal immune system and ENS are tightly intertwined. The mechanistic cooperation between these two systems begins in the early developmental stages – the functional constituents of the ENS mature concurrently with the immune system and with microbial colonization of the postnatal gut (Geuking *et al*., 2014; Kabouridis & Pachnis, 2015). Thus, the majority of enteric neurons and glial cells, as well as their connectivity, become fully formed under the direct influence of the developing microbiome. Peyer's patches, a vital element of the mucosal immune system, co‐mature with the ENS during this developmental period, under the same molecular signalling pathway (Patel *et al*., 2012). The immune system is thus highly involved in the many ENS‐modulated, CNS‐independent functions of the gut, including digestion and metabolism (Ma & Ma, 2019), gut motility (De Winter, 2010) and secretion of peptides and neurotransmitters (Khalil, Zhang & Engel, 2019).

The immune system is known to mediate processes in the CNS, with substantial evidence linking it to the induction of synaptic plasticity (Di Filippo *et al*., 2013). It is firmly established that increased systemic inflammation in later life contributes to decreased synaptic plasticity in the brain (Deleidi, Jãggle & Rubino, 2015), with pertinent evidence of an altered microbiome later in life (Nagpal *et al*., 2018) to support the hypothesis that the three phenomena are connected.

## V. NEUROTRANSMITTERS

### (1) Serotonin

The importance of neurotransmitters in the microbiome–gut–brain axis is compounded by the importance of neurotransmitters to a host of CNS (Lesch & Waider, 2012; Kraus *et al*., 2017) and ENS (Boesmans *et al*., 2008) processes and the ability of the microbiome to synthesise molecularly identical neurotransmitters that are biologically active in the host (Strandwitz, 2018). Additionally, the microbiome is known to mediate tryptophan metabolism by the host (O'Mahony *et al*., 2015). The neurotransmitter 5‐HT plays a vital role in both the CNS (Daubert & Condron, 2010) and ENS (Sasselli *et al*., 2012).

5‐HT is a functionally diverse neurotransmitter with extensive implications in neuroplasticity and modulation of a diverse range of functions (Daubert & Condron, 2010; Lesch & Waider, 2012; Lovelace *et al*., 2017). It is the most widely distributed transmitter in the brain, and its signalling pathways mediate not only homeostatic physiology, but also brain functionality, including sensory processing, cognitive control, emotional regulation, autonomic responses and motor activity, in a complex network of connections spanning the entire brain (Barton *et al*., 2008; Bennett & Maxwell, 2010; Lesch & Waider, 2012). Consequently, 5‐HT is a target of many physiological regulators, including modulators of gene transcription, neurotrophic peptides, and steroids, as well as psychotropic therapeutics, which impact the formation and activity of 5‐HT subsystems (Homberg *et al*., 2014). 5‐HT and BDNF are functionally linked. Examples of this functional interplay include the involvement of BDNF in the development and functionality of serotonergic neurons (Martinowich & Lu, 2008) and the co‐expression of BDNF and 5‐HT during heightened psychological arousal (Jiang *et al*., 2016).

The majority of the body's 5‐HT is contained within the gut (Yano *et al*., 2015); thus, the microbial communities colonizing the gut are hypothesized to exert an influence over its production and circulation, thereby affecting its downstream physiological roles (Clarke *et al*., 2013; Homberg *et al*., 2014; O'Mahony *et al*., 2015; Yano *et al*., 2015). 5‐HT is synthesized in two pathways. The first is from dietary tryptophan in enterochromaffin cells in the gut (Israelyan & Margolis, 2019), and the second is from albumin‐bound tryptophan that crosses the blood–brain barrier and is used for 5‐HT synthesis in the raphe nuclei of the brainstem (Lesch *et al*., 2012). The principal means of intraneuronal metabolism of 5‐HT is *via* oxidative deamination by monoamine oxidase resulting in formation of 5‐hydroxyindoleacetic acid (5‐HIAA) (Barton *et al*., 2008). Recent research supports microbial involvement in the metabolic processes pertaining to both of these metabolites (Waclawiková & El Aidy, 2018).

Preclinical studies have demonstrated that the GF condition is characterized by increased plasma tryptophan concentrations which can be normalized following microbial colonization of mice immediately post‐weaning (Clarke *et al*., 2013). Increased 5‐HT turnover, as indicated by the 5‐HIAA/5‐HT ratio, is also evident in the striatum of GF mice (Waclawiková & El Aidy, 2018). Other investigators have also reported elevations in plasma levels of both tryptophan and 5‐HT in GF mice compared to conventional animals (Wikoff *et al*., 2009). Interestingly, there may be a temporal effect of the colonization process. For example, one study reported that the elevated tryptophan concentrations in GF mice were reduced 4 days following the introduction of a microbiome but not at day 30 (El Aidy *et al*., 2012). Also of note is a recent study showing that GF rats have decreased hippocampal 5‐HT concentrations but exhibit stress‐induced elevation in both 5‐HT and 5‐HIAA, like conventional rats (Desbonnet *et al*., 2008). The impact of the gut microbiome on the CNS serotonergic system is not limited solely to microbiome‐deficient animals; administration of the probiotic *Bifidobacterium infantis* to rats resulted in reduced 5‐HIAA concentrations in the frontal cortex (El Aidy *et al*., 2012). Furthermore, there was also a marked increase in plasma concentrations of tryptophan and kynurenic acid in these animals. Additionally, the *Bifidobacterium*‐treated group exhibited reduced dihydrophenylacetic acid (DOPAC) and NA concentrations.

Clarke *et al*. (2013) showed that GF mice have impaired immune systems and heightened activity of the HPA axis but that sex differences exist in terms of BDNF and 5‐HT expression. BDNF expression was decreased in GF males, whereas 5‐HT, plasma tryptophan and 5‐HIAA levels were increased. Females did not exhibit significantly altered levels of these neurotransmitters. GF animals of both sexes exhibited a decreased kynurenine to tryptophan ratio (KYN/Trp) in plasma, which was restored to normal levels upon microbial colonization. The kynurenine pathway, which is heavily implicated in brain plasticity in health and disease, is responsible for catabolizing 95% of both CNS and peripheral tryptophan, with kynurenine being its primary catabolite. KYN/Trp is one of several ratios indicative of the pathway's functionality (Savitz, 2020). This neuromodulatory effect of *Bifidobacterium* is in line with reports of lowered NA levels in tricyclic antidepressant‐treated rats (Nakajima *et al*., 2012). Another study (Heijtz *et al*., 2011), which used behavioural testing to investigate the reduced anxiolytic behaviours of GF mice and the neurochemistry involved, found a significantly higher striatal turnover rate of NA, dopamine and 5‐HT in GF mice compared to SPF mice, but levels in the frontal cortex and hippocampus were unchanged.

In the ENS, 5‐HT secretion is mediated by the microbiome, and the activation of its 5‐HT_4_ receptor exerts neuro‐modulatory effects (Wood, 2001). De Vadder *et al*. (2018) showed that 5‐HT is necessary for adult ENS maintenance. Tryptophan hydroxylase (Tph) is the rate‐limiting enzyme in 5‐HT synthesis, and two different variants of this enzyme are used by enterochromaffin cells and neurons (Tph1 and Tph2, respectively), leading to 5‐HT synthesis in two distinct aggregations. De Vadder *et al*. (2018) also showed that *Tph1*‐deficient GF mice only exhibited a decrease in myenteric neuronal mass following colonization with a wild‐type microbiome, indicating that 5‐HT is crucial for healthy ENS development during early colonization of the gut. Additionally, conventional mice that were treated with irreversible Tph1 and Tph2 blockers showed decreased neuronal differentiation and less overall myenteric neuron functionality – factors which contribute to the efficacy of signal transduction and therefore induction of plasticity.

Selective serotonin‐reuptake inhibitors (SSRIs) are thought to exert their effects on gut 5‐HT in a manner that contributes to their anti‐depressant effects on the brain (Jones *et al*., 2020). Due to the role of the vagus nerve as a conduit between the gut, and consequently the microbiome, and the brain, McVey Neufeld *et al*. (2019) investigated the relationship between SSRI administration and vagal neuron firing rate, and the potential involvement of the microbiome in this bi‐directional pathway. Their results demonstrated that SSRI‐induced vagal neurostimulation is contingent upon signalling between the gut epithelium and afferent enteric neurons. Additionally, SSRI treatment altered the microbiome of the recipient mice, with the treatment group exhibiting reduced alpha diversity in comparison to the control group.

Taken together, these studies suggest the ability of the gut microbiome to influence the CNS and ENS serotonergic system profoundly, supporting our hypothesis that the gut microbiome is implicated in synaptic plasticity.

### (2) Dopamine

Dopamine acts on both excitatory and inhibitory synapses distributed throughout the brain, but a high density of dopaminergic receptors in specific areas has led to the recognition of several major dopaminergic pathways implicated in a host of brain functions and neuropsychiatric disorders (Pignatelli & Bonci, 2015; Aarts *et al*., 2017; Parashar & Udayabanu, 2017). In the gut, several types of dopamine receptors mediate vasoactive and homeostatic effects.

A study examining the relationship between the microbiome and alcohol‐related addictive behaviour in mice found a strong correlation between the striatal expression of the dopamine receptor messenger RNA (mRNA) in the brains of addiction‐prone mice and specific microbial alterations in the gut after alcohol exposure followed by a three‐month abstinence period (Jadhav *et al*., 2018). Dopamine 1 receptors (D1Rs), comprising the direct pathway that mediates reinforcement learning and award‐seeking behaviour, were observed at higher levels, whereas the aversive‐learning modulator D2R was observed in lower abundance. Dopamine receptors (Chen, Hopf & Bonci, 2010; Goto *et al*., 2010; Nakano *et al*., 2010) have been shown to contribute to induction of stimulus‐dependent plasticity in areas of the brain responsible for emotional regulation. Jadhav *et al*. (2018) showed that changes in dopamine receptor expression were concomitant with microbial alterations in several taxa, including Lachnospiraceae, *Syntrophococcus*, *Shuttleworthia*, *Gemella*, *Allobaculum*, and *Hydrogenoanaerobacterium*.

Another study in humans with and without attention deficit hyperactivity disorder (ADHD) combined 16S sequencing with functional magnetic resonance imaging (fMRI) to connect microbial differences and dopaminergic‐transmission alteration in the striatum. The results showed increased *Bifidobacterium* levels in ADHD patients, coupled with deficient ventral striatal responses in a reward‐anticipation fMRI test (Aarts *et al*., 2017).

Dopamine signalling is widespread in the ENS and is necessary for gut motility (Rao & Gershon, 2016). Xue *et al*. (2018) compared dopamine synthesis, dopaminergic neuron viability and correlated immune activity in conventional and antibiotic‐treated mice. Microbially deficient mice exhibited markedly reduced tyrosine hydroxylase (TH; the rate‐limiting enzyme for dopamine synthesis) mRNA in the gut, resulting in increased interferon‐gamma production, and exacerbation of autoimmune hepatitis. They also showed that dopamine inhibits cytokine production *via* its D1R. These patterns can affect plasticity as demonstrated by the interconnectedness of the CNS and the immune system, especially as it pertains to the onset of pathophysiological situations (D'Agostino *et al*., 2012; Fung *et al*., 2017).

Emerging evidence links the microbiome to the pathophysiology of Parkinson's disease (PD) (Picconi, Piccoli & Calabresi, 2012), with dopamine playing a major role in this process. The pathophysiology of this devastating disease is well described as neurodegeneration of dopaminergic cells in the substantia nigra, associated with accumulation of α‐synuclein aggregates in the cellular bodies (synucleinopathy). Kim *et al*. (2019) demonstrated the vagus‐dependent spreading of α‐synuclein from the gut to the brain, correlating with a loss of dopaminergic neurons and characteristic PD symptoms. Interestingly, significant synucleinopathy is also present in the ENS. These findings, along with the fact that with the onset of PD, gastrointestinal symptoms often precede motor symptoms by several years (Elfil *et al*., 2020), may lead to a paradigm shift as to the true origin of PD. *Helicobacter pylori* has been shown to be overrepresented among PD patients and exacerbates symptoms by interfering with the metabolism of levodopa, the primary pharmaceutical administered to PD patients (Contin & Martinelli, 2010). Dysbiosis and small intestinal bacterial overgrowth (SIBO) are also characteristic of PD (Tan *et al*., 2014). PD patients’ bacterial profile indicates a reduction of Prevotellaceae, as well as a direct correlation between abundance of Enterobacteriaceae and severity of some motor symptoms (Parashar & Udayabanu, 2017).

In a murine model of PD (CDNF‐knockout mice), cerebral dopamine neurotrophic factor (CDNF) restored dopaminergic function in the nigrostriatal area of the brain (Lindahl *et al*., 2020). Lindahl *et al*. (2020) demonstrated that CDNF protects against enteric neuropathy occurring in old age, specifically in the submucosal plexus, which corresponds to enteric pathologies present in human PD patients. Interestingly, the CNS concentration of dopaminergic neurons in the CDNF‐knockout mice used for this study was unaltered.

## VI. THE MICROBIOME AND NEURO‐IMMUNITY

The complex mechanisms of neuro‐immune regulation of gut physiology are numerous; therefore, a detailed exploration is beyond the scope of this review. We briefly review the main pathways by which neuro‐immune regulation is carried out, with evidence of microbial intervention on several aspects of this functional relationship.

The intestinal neuro‐immune interface encompasses (Yoo & Mazmanian, 2017) up to 80% of the body's immune cells, more than 100 million neurons and upwards of 100,000 synapses between enteric nerves and extrinsic (Uesaka *et al*., 2016) nerve endings from the vagus and pelvic nerves. While the ENS can function completely autonomously (Sasselli *et al*., 2012), the structural interface, with extrinsic sympathetic and parasympathetic nerves, allows the gut to send mechanical and chemosensory information back to the brain *via* the vagus nerve (Bonaz, Sinniger & Pellissier, 2017), and the brain to modulate gut functionality (Cryan *et al*., 2019) that requires nervous signal transmission, such as gut motility, contractility and vasodilation (Sasselli *et al*., 2012). Immunological structures (Yoo & Mazmanian, 2017) extend to the gut epithelium with the express purpose of sensing pathogenic material and enlisting an appropriate immune response. The myriad interactions between the microbiome and the enteric nervous system are clearly demonstrated in GF mice (Zheng *et al*., 2020). GF mice exhibit greatly reduced intra‐epithelial lymphocyte and immunoglobulin A (IgA) antibodies, an absence of T helper 17 (Th17) cells and an imbalance of Th1/Th2 cells (Kim, 2018). These phenomena indicate the involvement of the microbiome in both the innate and adaptive immune systems. The structural proximity of immune and nervous system structures gives rise to a functional overlap, compounded by molecular cross‐talk between the two systems (Khalil *et al*., 2019). Immune mediators are known to influence enteric neuronal activity, and neuroactive molecules affect the function of immune cells (Khalil *et al*., 2019). Macrophages, dendrites and T cells are activated by Ach, 5‐HT and vasoactive intestinal polypeptides secreted by enteric nerves, and cytokines, proteases and opioids released by the immune cells in turn modulate the sensitivity and transcriptional profile of neurons (Khalil *et al*., 2019). This is especially evident in gastrointestinal pathologies (Fung *et al*., 2017) which exhibit comorbidities of heightened immune responsivity and increased inflammation alongside electrical impairments in the gut and disturbances in perception of visceral pain. There is a growing body of evidence linking the microbiome to the onset and severity of inflammatory bowel disease (Ocansey *et al*., 2020). GF mice have a greater susceptibility for gastrointestinal dysfunction, and certain bacteria (Glassner, Abraham & Quigley, 2020) have been correlated to genetic mutations affecting epithelial defence against pathogens, mucosal barrier integrity, inflammatory gene expression and regulatory T‐cell differentiation (Schirmer *et al*., 2016; Kim, 2018; Lazar *et al*., 2018; Zheng *et al*., 2020).

*Lactobacillus rhamnosus* JB‐1 (JB‐1) (Liu *et al*., 2020) has been shown to require regulatory T cells for its role in mediating anxiolytic and depressive behaviours. Moreover, antibody depletion of CD25^+^ T regulatory (Treg) cells markedly reduced the beneficial behavioural effects of JB‐1. JB‐1 has been shown to exert mediating effects on anxiolytic and depressive behaviours in a vagus‐dependent manner (Perez‐Burgos *et al*., 2014), and it has also been shown to reduce GABA receptor expression (Bravo *et al*., 2011) in specific brain areas known to undergo extensive learning‐ and emotional‐regulation‐related plasticity. These multifaceted effects of JB‐1 exemplify the intricacy of the microbiome–gut–brain axis, with the multitude of constituents involved in its underlying mechanisms.

Additionally, the HPA axis also plays a vital role in the neuro‐immune regulation of the gut. Part of the limbic system, the HPA axis is the primary regulator of the stress response and is sensitive to a host of systemic molecules, including pro‐inflammatory cytokines released from the gut. In turn, corticotrophin releasing factor (CRF) from the hypothalamus triggers adrenal adrenocorticotropic hormone (ACTH) release and subsequent cortisol secretion. Aside from metabolically affecting most organ systems in the body, cortisol has been specifically implicated in mental disorders and stress‐related gastrointestinal and immune disorders (Wiley, Higgins & Athey, 2016). The microbiome has been shown to modulate the HPA axis extensively (Frankiensztajn *et al*., 2020). GF mice exhibit decreased anxiety and increased stress responsivity in conjunction with increased ACTH and cortisol levels. *Bifidobacterium pseudocatenulatum* (Moya‐Pérez *et al*., 2017) has been shown to prevent stress‐induced increases of corticosterone and hypothalamic catecholamines in mice, and also to cause a substantial decrease in stress‐induced inflammatory markers.

The vagus nerve (Bonaz *et al*., 2017), acting as a conduit between the ENS and the brain, synapses in the solitary tract nucleus, which sends projections to most regions of the brain, including the hypothalamus, further cementing the interconnectedness between the various branches of the microbiome–gut–brain axis. Evidence of vagus‐dependent microbial influence on the CNS includes (Fülling, Dinan & Cryan, 2019) *Campylobacter jejuni*'s ability to increase anxiolytic behaviours, as well as *Fos* transcription factor (belonging to the IEG family) activity in vagal afferents and the solitary tract nucleus, the prevention of beneficial effects of *L. rhamnosus* JB‐1 in vagotomized mice (Liu *et al*., 2021) and the rescuing of social deficit by *L. reuteri* in animal models of ASD. The vagus nerve is also among the hypothesized channels by which bile acids (BAs) (Wu *et al*., 2020) and short‐chain fatty acids (Goswami, Iwasaki & Yada, 2018) exert their influence on the CNS. Short‐chain fatty acids are the product of microbial fermentation of dietary fibre and are thought to modulate a wide range of brain activities *via* immune, endocrine, vagal and other humoral pathways (Dalile *et al*., 2019). BAs are metabolically active hormonal mediators, known to be regulated by the microbiome (Monteiro‐Cardoso, Corlianò & Singaraja, 2021). BAs are synthesised in the liver and are systemically active, with BA receptors widely distributed in the brain as they are capable of crossing the blood–brain barrier. Hypothesized to act *via* the vagus nerve, BAs have been implicated in severe neuropsychiatric diseases (Sandhu *et al*., 2017).

## VII. MICROBIAL EFFECTS ON ELECTROPHYSIOLOGY

The most direct evidence of the involvement of the microbiome in the regulation of synaptic plasticity comes from electrophysiological recordings of synaptic activity under different conditions of microbial treatment or after modulation of the host microbiome. Detecting changes in paired‐pulse facilitation is one mechanism to test effects on synaptic function.

Paired‐pulse facilitation (PPF) (Hu *et al*., 2013) refers to the effect wielded by a stimulated presynaptic cell on the magnitude of the second current emitted by a postsynaptic cell. This indicates a transient enhancement of transmission capacity following signal transduction in that specific synapse, a type of use‐dependent, short‐term plasticity. PPF is measured by plotting field excitatory postsynaptic potentials (fEPSPs) as a function of time elapsed between two consecutive action potential firings, and is hypothesized to be crucial for the induction of LTP (Tahmasebi *et al*., 2016).

One study (Davari *et al*., 2013) correlating microbial influence with EPSP transmission in the hippocampus studied diabetic and control rats after receiving probiotic treatment and undergoing a spatial learning test. The results showed that a probiotic cocktail composed of *Lactobacillus acidophilus*, *Bifidobacterium lactis* and *Lactobacillus fermentum* improved the learning task performance of both groups (diabetics and controls) and considerably rescued hippocampal EPSP response in the diabetic group. Also, the diabetic–probiotic group exhibited the greatest increase in LTP, which is significant due to the known phenomenon of diabetes‐induced learning and memory deficits (Zilliox *et al*., 2016).

Sgritta *et al*. (2019) utilized the AMPAR/NMDAR ratio in dopaminergic neurons to measure the effects of *L. reuteri* on social stimuli‐induced synaptic potentiation in a mouse model of ASD with known impairments to oxytocinergic transmission. AMPARs (Chater & Goda, 2014) play a crucial, dual role in the induction of synaptic plasticity. The opening of AMAPRs allows an influx of cations which triggers action potential firing and enlists calcium‐dependent signalling cascades (Nanou & Catterall, 2018; Kornijcuk *et al*., 2020) which catalyse long‐lasting changes in the structure and functionality of the neuron. NMDARs play an important role in AMPAR‐mediated plasticity (Moosmang, 2005; Hunt & Castillo, 2012), and the ratio of the two receptors’ expression levels mediates trends in neurotransmission that are linked to the induction and long‐term maintenance of plasticity (Rao & Finkbeiner, 2007). The results of this study showed that the AMPAR/NMDAR ratio, while similar at baseline levels between the ASD model and controls, failed to increase and induce LTP in the ASD mice in response to social stimuli alone. Additionally, *L. reuteri* administration rescued social deficits in the ASD mice in a vagus‐dependent manner, as confirmed by vagotomized controls. These findings suggest a direct effect of the microbiome on CNS transmission.

The major mechanisms through which the microbiome may affect LTP remain elusive. One possible mechanism is through the production of specific metabolites which can cross the blood–brain barrier and affect neuronal function and synaptic plasticity. Govindarajulu *et al*. (2020) set out to determine the potential effects on LTP induction by the microbiome‐dependent metabolite trimethylamine N‐oxide (TMAO). TMAO is a by‐product of microbial metabolism of nutrients, and has been implicated (Li *et al*., 2018) in age‐related cognitive impairments in mice. The rationale of this study was that a decline in LTP induction, influenced by increased levels of TMAO, is the mechanism behind old‐age cognitive deficits. The results of this study showed reduced fEPSP measurements and PPF in hippocampal slices incubated with TMAO, compared with controls. Western blot analysis revealed reduced expression of AMPAR subunits, which may account for the observed LTP deficits, as well as impaired basal synaptic transmission. This study provides evidence for a role of microbial metabolites in the regulation of synaptic plasticity.

The implications of direct microbial influence on electrophysiological events necessary for the induction and long‐term maintenance of synaptic plasticity are vastly important for understanding the mechanisms of the microbiome–gut–brain axis.

## VIII. GENETIC EXPRESSION, SIGNALLING CASCADES AND NEUROTROPHIC FACTORS

One of the mechanisms responsible for synaptic plasticity is dynamically regulated gene expression and associated protein synthesis (Glanzman, 2010; Korb & Finkbeiner, 2011; Gipson, Kupchik & Kalivas, 2014; Berger *et al*., 2017). Patterns of gene expression vary widely, depending on the brain region undergoing the plasticity‐induced changes and the type of plasticity process taking shape, and these mechanisms have been studied extensively in humans and animal models. IEGs (Coppens *et al*., 2011) are heavily involved in plasticity induction due to their fast transcription that does not require *de novo* protein synthesis (Okuno, 2011). Prominent IEGs include the *Arc*, *Fos* and *Egr* gene families. Recently, researchers have begun to uncover connections between the gut microbiome and gene expression associated with plasticity (Dinan & Cryan, 2017).

The amygdala is a key element of the limbic system, responsible for the processing and integration of emotional‐related stimuli. Amygdala emotion‐induced learning and its consolidation into behavioural adaptations is realized *via* synaptic plasticity. Behavioural testing involving fear conditioning and socialization paradigms has become standard practice in animal studies investigating plasticity‐related functionality of the amygdala under different circumstances, especially in emotional disorders including post‐traumatic stress disorder, affective disorders and anxiety (Malter Cohen *et al*., 2013).

Hoban *et al*. (2018) conducted a preliminary examination of the brains of GF mice before subjecting them to a fear‐conditioning behavioural test. The researchers found that the mice exhibited a basally modified pattern of transcription, compared to conventionally raised animals, specifically as it pertains to the expression of IEGs including *Fos*, *Egr2*, *Fosb* and *Arc*. Such results support a neuronal basis for the well‐documented behavioural and emotional phenotypic alterations presented by GF animals. Also altered was the expression of genes necessary for neuronal functionality, synaptic transmission and developmental processes in the CNS. Following the behavioural test, GF mice did not appear to learn fear responses as well as their conventional counterparts, and subsequent genome‐wide transcriptome profiling revealed a total of 133 differentially expressed genes related to plasticity.

Another study (Stilling *et al*., 2018) focusing on the amygdala's role in social behaviour, and employing similar methods as above (investigation of brains of GF animals, behavioural testing and RNA sequencing), found significant differences in alternative splicing, intracellular signalling and phosphorylation, specifically in the mitogen‐activated protein kinase (MAPK) signalling pathway, which is heavily involved in regulation of synaptic plasticity (Falcicchia *et al*., 2020). These results suggest a baseline hyperactivity in the amygdalae of GF mice. GF mice did not perform as well, on average, in the behaviour test compared to GF mice colonized with a healthy microbiome at weaning or to conventional mice. Splicing activity was improved in GF mice following social interaction and was even more pronounced in ex‐GF mice, which displayed a behavioural response strongly resembling that of conventional mice. The expression profile of serotonergic receptor genes was not significantly different between GF and conventional animals of either sex. These results seem to suggest a mechanistic correlation between a healthy microbiome and functional integrity of the amygdala in inducing correct behavioural patterns in response to social stimuli.

Another study (Davari *et al*., 2013) examined the effects of probiotic treatment (*L. acidophilus*, *Bi. lactis* and *L. fermentum*) on the behavioural and electrophysiological aspects of diabetes‐induced learning and memory deficits in rats. Results of this study showed that diabetic animals that received probiotics exhibited markedly improved spatial memory, facilitated by an improved ability of the brain to perform LTP, a key element of synaptic plasticity.

A third study (Zolfaghari, Rabbani Khorasgani & Noorbakhshnia, 2020) measuring hippocampal CaMKII‐α and tumor necrosis factor (TNF‐α) expression showed that probiotic treatment with *L. rhamnosus*, *L. reuteri* or *L. plantarum* successfully impeded neuroinflammation‐induced memory deficits in mice. CaMKII‐α is one of the major factors responsible for induction of LTP. Lipopolysaccharide (LPS) is an endotoxic, neurotoxic and inflammatory molecule found in gram‐negative bacterial membranes, capable of stimulating the expression of TNF‐α and interleukins IL‐1β and IL‐6 (Zweigner, Schumann & Weber, 2006). The results of this study showed that *L. rhamnosus* ameliorated LPS‐induced impairment of hippocampal CaMKII‐α gene expression. Additional studies (Lim *et al*., 2017; Lee, Lim & Kim, 2018) highlight the ability of *Lactobacillus* to correct such impairments through administration of *L. johnsonii* CJLJ103 (LJ) to mice. LJ corrected two detrimental effects caused by LPS: nuclear factor kappa B (NF‐κB)‐induced hippocampal neuroinflammation and reduced BDNF expression.

Synaptic plasticity is a multi‐factorial process, enabled by differential gene expression and a consequent web of signalling cascades. Although some genes, like *Arc* and *Fos*, are directly linked to induction of plasticity, other markers of plasticity are activated indirectly, causing plasticity to occur downstream, for example *via* neurotransmitter release or BDNF (Leal *et al*., 2014; Falcicchia *et al*., 2020). Any functional modifications to the molecular machinery of the neuronal environment that lead to the induction of plasticity events can be traced back to altered gene expression and the ensuing protein synthesis, either of which can serve to indicate and quantify the processes taking place (Glanzman, 2010; Kotaleski & Blackwell, 2010).

BDNF is a growth factor that is abundantly expressed in all major brain regions and is implicated in a wide array of supportive and survival‐oriented functions, as well as higher cognitive functions such as learning, memory and emotional regulation (Cunha, Brambilla & Thomas, 2010; Lu *et al*., 2014; Loprinzi & Frith, 2019). Alterations in the expression or signalling of BDNF are implicated in the pathophysiology of mental disorders such as depression (Yu & Chen, 2011) and schizophrenia (Favalli *et al*., 2012), execution of motor functions (He *et al*., 2013) and LTP and consequent memory formation (Cunha *et al*., 2010; Loprinzi & Frith, 2019).

BDNF is crucial for the healthy development of the CNS, and its absence results in a multifaceted phenotype of cognitive and mental disorders, as evidenced from animal studies (Taliaz *et al*., 2010; Coppens *et al*., 2011; He *et al*., 2013; Jiang *et al*., 2016). Numerous studies examining the effects of the microbiome on CNS development show a clear correlation between a GF condition or antibiotic‐induced microbiome ablation with reduced BDNF levels in conjunction with aberrant cognitive and behavioural phenotypes. BDNF is tightly coupled with a range of processes occurring in synapses, particularly induction of plasticity, due to its involvement in many downstream signalling cascades and its presence in both inhibitory and excitatory synapses in all brain regions (Cunha *et al*., 2010; Favalli *et al*., 2012; Lu *et al*., 2014). Thus, it is probable that the microbiome plays an integral part in early developmental processes in the brain as it pertains to the expression of cardinal molecules such as BDNF.

Stilling *et al*. (2015) found enrichment of the transcription factors *Fos*, *Egr2* and *Nr4a1*, as well as the plasticity‐related genes *Arc* and *Homer1*, in mice immediately following socialization in a behavioural test, indicating that plasticity activation is increased in brain areas that have been stimulated. The importance of examining the IEG expression profile concurrently with BDNF levels is that it was shown to be differentially expressed during BDNF‐dependent plasticity events (Coppens *et al*., 2011).

Also upregulated in socialized mice was the neuronal activity‐induced MAPK signalling pathway, paralleling the results of previous studies (Verma *et al*., 2016). A real‐time, quantitative, reverse transcription polymerase chain reaction (qRT‐PCR) was used to determine BDNF levels in GF mice as an indicator of cyclic AMP response element‐binding protein (CREB) expression. CREB is a crucial modulator of neuronal activity and is an important downstream effector of BDNF expression. Neufeld *et al*. (2011) showed similar results in a study that compared gene expression in the brains of GF and SPF mice, assisted by targeted neuronal stimulation provided by behavioural testing. GF mice exhibited anxiety‐like behaviour in the elevated plus maze, and their expression profile included decreased amygdaloid mRNA expression of NMDAR subunit NR2B, increased hippocampal BDNF levels and depleted levels of 5‐HT receptor 1A (5‐HT‐1A).

Correct gene expression in the CNS during neonatal development is critical for healthy maturation of countless brain functions, as evidenced in cases where the smallest alteration in gene expression results in complicated syndromes that affect an organism's entire well‐being (Veenstra‐VanderWeele *et al*., 2012; Zhan *et al*., 2014). Efforts to understand the impact of a healthy microbial transfer between mother and offspring on the latter's CNS development are aided by preclinical studies involving GF mice (Jenmalm, 2017; Torres *et al*., 2020). These studies can highlight the phenotypic differences in animals maturing with or without an intact microbiome. One such study (Lu *et al*., 2018) transferred the microbiome of preterm human infants to GF mice in order to pinpoint neuronal markers that were altered in congruence with the affected phenotype. Mice that received the preterm microbiome displayed poor development and a decreased expression profile of the neuronal markers *NeuN* and neurofilament‐L and myelination marker myelin basic protein (MBP). As is clinically observed in preterm infants, the ‘induced preterm’ mice exhibited increased neuroinflammation as measured by increased *Nos1* and *Igfbp3* brain mRNA expression, altered insulin‐like growth factor 1 (IGF‐1) signalling and decreased circulating insulin‐like growth factor binding protein 3 (IGFBP3). A different study (Chen *et al*., 2017) examined the microbial influence on hippocampal microRNA (miRNA) and mRNA expression. Utilizing behavioural tests to compare GF and SPF mice, followed by RNA sequencing, the researchers showed that the GF mice exhibited decreased anxiety‐like behaviour, supported by a deficient RNA expression profile, which was partially restored following colonization with SPF microbiome. The genetic significance of the RNA expression alterations lies in their importance in metabolic, cellular binding and guiding and molecular processes as they pertain to neuronal functionality. GF mice, repeatedly shown to exhibit reduced anxiolytic responses, were neurochemically examined in several major brain areas. *In situ* hybridization revealed reduced levels of *NGFI‐A* and BDNF mRNA. NGF is abundantly present in the hippocampus and takes part in HPA activity in an IEG‐inducible manner. NGFI‐A expression is implicated in situations related to stress, abnormal electrical activity such as seizures and following the induction of synaptic plasticity in the form of LTP (Olsson *et al*., 1994). This study also showed, by qPCR, a higher level of synaptophysin and postsynaptic density protein 95 (PSD‐95) (Ocansey *et al*., 2020) in the striata of GF mice (Olsson *et al*., 1994). Synaptophysin, expressed in most CNS neurons and particularly in neuroendocrine cells, is necessary for synaptic vesicle maturation and is used as a secondary indicator of developmental synaptogenesis (Hami *et al*., 2016). Also necessary for developmental processes in excitatory synapses is PSD‐95 (Prange *et al*., 2004). Microbial influence on proper CNS functionality is often caused by a perturbation to the normal microbiome as a result of infection or another microbial imbalance leading to an exacerbated presence of a certain pathogen. Gareau *et al*. (2011) introduced *Citrobacter rodentium* to GF and conventional mice and tested memory impairment in the presence or absence of an added stressor in the form of water avoidance stress (WAS). Following introduction to the aversive WAS stimulus the *C. rodentium*‐infected mice exhibited decreased hippocampal BDNF and c‐Fos levels, which were ameliorated by probiotic treatment. Interestingly, it was only the combination of *C. rodentium* and WAS that produced the alterations in BDNF and c‐Fos expression.

Due to its highly diverse functionality, BDNF is necessary for a number of brain processes, both homeostatic and plastic in nature. Evidence linking the modulation of BDNF to the microbiome signifies a step towards unravelling the mechanistic relationship between the microbiome and synaptic plasticity.

## IX. SYNTHESIS

The existence of a microbiome–gut–brain axis is overwhelmingly supported, with correlations between the microbiome and a wide spectrum of cognitive, emotional and behavioural phenotypes, in health and disease, as well as homeostatic brain functionality. Currently, the vast potential of the microbiome as a diagnostic and therapeutic tool is undercut by a dearth in the mechanistic understanding of how the microbiome exerts its influence on the host's brain; however, some promising evidence is emerging. Probiotics (live, beneficial microorganisms introduced to the host), prebiotics (non‐digestible dietary fibre that serves as an energy source for the host's resident microbiome) and synbiotics (a combination of the two) have been increasingly used in a clinical setting by a variety of medical fields, exhibiting salubrious health effects, including normalization of neuropsychiatric symptoms (Moosmang, 2005; Kali, 2016; Cepeda, Katz & Blacketer, 2017; Mörkl *et al*., 2020; Morshedi, Saghafi‐Asl & Hosseinifard, 2020; Skott *et al*., 2020).

The microbiome interacts extensively with the mucosal immune system and the enteric nervous system, which operate synergistically with effects spanning many organ systems, particularly the brain. Microbial metabolites affect the brain through several branches of the microbiome–gut–brain axis *via* the parasympathetic vagus nerve that connects the ENS the CNS and the systemic immune system or the HPA axis which are interconnected as well. Synaptic plasticity is one of the most fundamental processes occurring in the brain, implicated in learning and memory consolidation. Dysfunctions in induction of synaptic plasticity are implicated in severe neuropsychiatric disorders. The mechanisms of plasticity are highly complex and include many types of receptors and their trafficking across membranes, signalling and cell adhesion molecules. The presynaptic cell is stimulated by a neurotransmitter which in turn activates ionic channels on the presynaptic cell, altering its membrane potential and ionic permeability, thereby affecting its ability to fire an action potential. ENS plasticity is emerging as a vastly important research target due to the complex interconnectedness between the CNS and ENS, as well as the latter's substantial involvement in various physiological and pathological phenomena. Moreover, the ENS shares many similarities with the CNS – from its neuronal circuitry to the neurotransmitters activating it.

The microbiome has been shown to influence several plasticity inducers as well as homeostatic maintenance, through various neurotransmitters, BDNF, and expression of relevant genes. While research into the effects of the microbiome on synaptic plasticity is shifting to the ENS, very few studies have investigated the interplay between plasticity in the ENS and the CNS, under the influence of the microbiome and its metabolites (Table 1). While conducting electrophysiological studies on GF mice is complicated by setting up adequate equipment within a sterile environment, we believe that incorporating such examinations into studies involving SPF or conventional mice is necessary. Electrophysiological studies of an animal's ENS as well as CNS are needed, together with measurements of plasticity‐related markers in both the CNS and the gut. Moreover, incorporating metabolomics analyses into microbiome studies aimed at unravelling the mechanistic connections between metabolites and synaptic plasticity will provide a more accurate picture of which metabolites are significantly altered, in conjunction with insights from electrophysiological studies. Additionally, we strongly believe in the big‐data approach of modelling such complex systems and interactions. There is evidence that computational‐oriented mapping of all the different molecular constituents of plasticity, the vast array of microbial metabolites and other physiological markers from the host, might yield a better understanding of how all these different elements fit together (Eetemadi *et al*., 2020; Mäki‐Marttunen *et al*., 2020).

**Table 1:** Potential microbial effectors of synaptic plasticity

	Target nervous system	Hypothesized plasticity‐related mechanism affected
*Lactobacillus reuteri* (LR)	ENS	Calcium‐dependent channels in a subpopulation of enteric sensory neurons, implication in intestinal‐disorder derived pain (Kunze *et al*., 2009). LR probiotic‐treated mice exhibited reduced capsaicin‐induced excitatory response, accompanied by decreased expression of the nociceptive signal‐transmitting TRPV1 (Perez‐Burgos *et al*., 2015). Probiotic administration rescued social deficits in ASD mice in a vagus‐dependent manner.
*Bifidobacterium infantis*	CNS	Probiotic administration led to reduction of the serotonin intermediate 5‐HIAA in the frontal cortex, together with plasma increases of tryptophan, kynurenic acid, dihydroxyphenylacetic acid and noradrenaline (El Aidy *et al*., 2012).
Lachnospiraceae, *Syntrophococcus*, *Shuttleworthia*, *Gemella*, *Allobaculum*, and *Hydrogenoanaerobacterium*	CNS	Changes in dopamine receptor expression, known to contribute to induction of stimulus‐dependent plasticity in areas of the brain responsible for emotional regulation (Jadhav *et al*., 2018).
*Bifidobacterium*	CNS	Deficient ventral striatal responses in a reward anticipation fMRI test (Aarts *et al*., 2017).
Enterobacteriaceae	CNS	Correlated with severity of Parkinson's symptoms (Parashar & Udayabanu, 2017).
*Lactobacillus rhamnosus* JB‐1 (JB‐1)	CNS	Immune‐mediated modulation of anxiolytic and depressive behaviours (Liu *et al*., 2020). Affects anxiolytic and depressive behaviours in a vagus‐dependent manner (Perez‐Burgos *et al*., 2014), and has also been shown to reduce GABA receptor expression in specific brain areas known to undergo extensive learning and emotional‐regulation related plasticity (Bravo *et al*., 2011). *L. rhamnosus* ameliorated LPS‐induced impairment of hippocampal CaMKII‐α gene expression.
*Bifidobacterium pseudocatenulatum*	CNS	Prevention of stress‐induced HPA axis activation and consequent increases of corticosterone and hypothalamic catecholamines (Moya‐Pérez *et al*., 2017).
*Campylobacter jejuni*		Increase of anxiolytic behaviours, as well as Fos activity in vagal afferents and the solitary tract nucleus (Fülling *et al*., 2019).
*Lactobacillus acidophilus*, *Bifidobacterium lactis* and/or *Lactobacillus fermentum*	CNS	Probiotic cocktail led to improvement in learning task performance, as well as rescue of hippocampal EPSP response and markedly improved spatial memory, facilitated by an improved ability of the brain to undergo LTP (Davari *et al*., 2013).
*L. rhamnosus*, *L. reuteri* and/or *L. plantarum*	CNS	Probiotic administration impeded neuroinflammation‐induced memory deficits in mice (Zolfaghari *et al*., 2020).
*Lactobacillus johnsonii* CJLJ103		Probiotic administration of *L. johnsonii* CJLJ103 (LJ) to mice. LJ corrected two detrimental effects caused by LPS: NF‐κB‐induced hippocampal neuroinflammation and reduced BDNF expression (Lee *et al*., 2018)

5‐HIAA, 5‐hydroxyindoleacetic acid; ASD, autism spectrum disorder; BDNF, brain‐derived neurotrophic factor; CaMKII‐α, calcium/calmodulin‐dependent protein kinase type II subunit alpha; CNS, central nervous system; ENS, enteric nervous system; EPSP, excitatory postsynaptic potential; fMRI, functional magnetic resonance imaging; Fos, transcription factor belonging to the immediate early gene family; GABA, gamma aminobutyric acid; HPA, hypothalamus–pituitary–adrenal axis; LPS, lipopolysaccharide; LR, *Lactobacillus reuteri*; LTP, long‐term potentiation; NF‐κB, nuclear factor kappa‐light‐chain‐enhancer of activated B cells; TRPV1, transient receptor potential vanilloid 1.

The objective of this review was to highlight the multi‐factorial involvement of the microbiome with the vastly important process of synaptic plasticity, and to emphasize the need for a research paradigm shift, where the pathophysiology of complex neuropsychiatric and neurodegenerative disorders is involved (Table 1). We believe that studying the microbiome–gut–brain axis should henceforth include a significant neuroscience‐driven research methodology. In‐depth explorations into the intricate processes involved with synaptic plasticity should put equal emphasis on the microbial and the neuronal–electrophysiological aspects of the process. Indeed, they should focus on the contribution of the microbiome and its metabolites, given their undisputed importance to cognitive, emotional and behavioural aspects of the host. Such processes have been shown to depend heavily on successful induction and maintenance of synaptic plasticity.

## X. CONCLUSIONS

1. The microbiome has been unequivocally shown to affect the mental, emotional and cognitive functionality of the host, including both phenotypic manifestations and neurophysiological aspects.
2. Synaptic plasticity is a cardinal neurophysiological process occurring in both the CNS and ENS. It is responsible for the incorporation of long‐term functional changes to synaptic connectivity and structural integrity, and consequently, neuronal transmission. The consolidation of such plastic changes requires a host of molecular processes to occur, including alterations in gene expression and receptor trafficking, rapidly regulated signalling cascades, secretion of neurotransmitters and neurotrophic factors and more.
3. The microbiome has been shown to influence various different neurophysiological aspects of synaptic plasticity, with direct correlations established between microbial perturbations and neurotransmitter secretion, immediate early gene expression, neuronal surface receptor trafficking, neurophysiological properties affecting neuronal transmission and more. These diverse effects of the microbiome on the different mechanisms underlying synaptic plasticity suggest that the influence of the microbiome on the mental, cognitive and emotional wellbeing of the host is achieved by affecting synaptic plasticity directly and indirectly, thereby allowing for long‐lasting changes to take place.
